# Bilateral Subinternal Limiting Membrane Crystalline Deposits Secondary to Terson Syndrome

**DOI:** 10.1155/2024/8225960

**Published:** 2024-01-17

**Authors:** Anfisa Ayalon, Eran Greenbaum, Lily Okrent Smolar, Alexander Rubowitz

**Affiliations:** ^1^Department of Ophthalmology, Meir Medical Center, Kfar Saba, Israel; ^2^Sackler Faculty of Medicine, Tel Aviv University, Tel Aviv, Israel

## Abstract

**Background:**

We report the case of bilateral, subinternal limiting membrane crystalline deposits in a patient with Terson syndrome, describe the possible pathogenesis, and highlight management. *Case Presentation*. A 24-year-old male with a history of traumatic massive parenchymal and subdural frontal hemorrhage presented to our clinic seven months after a motor vehicle accident, prolonged hospitalization, and rehabilitation, complaining of decreased vision in both eyes. The Snellen visual acuity was 1/60 in the right eye, and 6/60 in the left eye. Fundus examination showed an organized white vitreous hemorrhage in both eyes with almost no view of the retina. The anterior segments were normal. He underwent a 25-gauge pars plana vitrectomy in both eyes. During the surgery, golden crescent-shaped sediment consisting of small crystals was observed under the internal limiting membrane in both eyes: anterior to the inferior temporal vascular arcade in the right eye and posterior to it in the left eye. Internal limiting membrane (ILM) peeling after staining with ILM-blue dye was performed in the left eye, where the finding involved the macula. One year after the surgery, visual acuity significantly improved to 6/8.5 on the right and 6/6 on the left. Epiretinal membrane formation was observed in the right eye, where ILM peeling was not performed.

**Conclusion:**

Subinternal limiting membrane crystalline deposit finding is a rare condition. Consider performing internal limiting membrane peeling and sediment removal in cases with macular involvement. In cases where crystals are concentrated outside of the macula, follow-up may be considered.

## 1. Introduction

Terson syndrome (TS) is characterized by intraocular hemorrhage secondary to subarachnoid hemorrhage. A number of theories exist regarding pathogenesis; it is possible that high intracranial pressure transmitted along the optic nerve sheath causes venous congestion and rupture of retinal vessels [1]. In cases due to traumatic head injury, TS is associated with a significantly higher mortality rate, and fundus examination is recommended for all patients with subarachnoid hemorrhage [2].

Vitreous hemorrhage is the most common finding in TS; however, concurrent hemorrhages (subhyaloid, subinternal limiting membrane, intraretinal, and/or subretinal) have all been described [1].

We describe the novel finding of subinternal limiting membrane (sub-ILM) synchysis scintillans–like finding in a patient with TS associated with a long-standing vitreous hemorrhage.

## 2. Case Presentation

A 24-year-old male presented to the emergency room after a motor vehicle accident with left-sided parenchymal and subdural frontal hemorrhage and a Glasgow Coma Scale score of three. He underwent prolonged hospitalization in the neurosurgery intensive care unit, with intubation and decompressive craniotomy followed by long-term rehabilitation. Seven months after the accident, the patient was able to complain of decreased vision in both eyes and was referred to our emergency department. On exam, the Snellen visual acuity in the right eye was 1/60 and in the left eye was 6/60. Anterior segments and intraocular pressure were normal. Fundus examination showed an organized white vitreous hemorrhage in both eyes over the posterior pole with no view of the fundal retinal details, while the peripheral retina was attached with no pathology.

**Figure 1:**
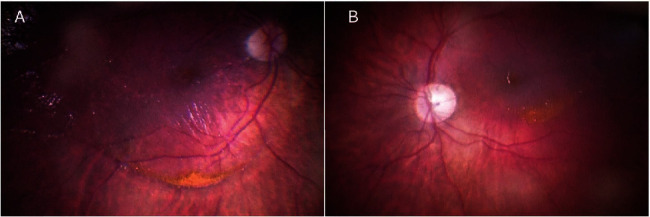
Intraoperative color picture of the posterior pole after core vitrectomy and posterior vitreous detachment inducement. (A) Right eye. Note: sub-ILM golden crescent-shaped deposits consisting of small crystals anterior to the inferior temporal vascular arcade, a “ring” sign secondary to detached ILM and ILM striae. (B) Left eye. Note: the similar sub-ILM crescent-shaped deposits with macular involvement and a “ring” sign.

**Figure 2:**
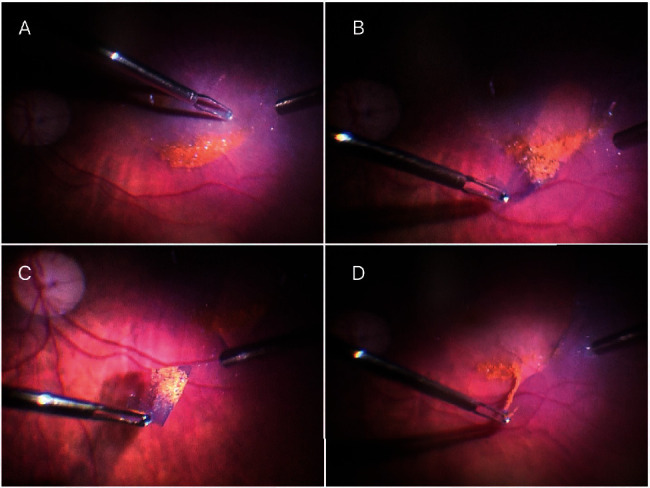
Intraoperative color picture of the left eye during ILM peeling after staining with ILM-blue dye (A, B). Note: the characteristic reflectivity of the golden crystals (C, D) which were firmly attached to ILM and retina.

**Figure 3:**
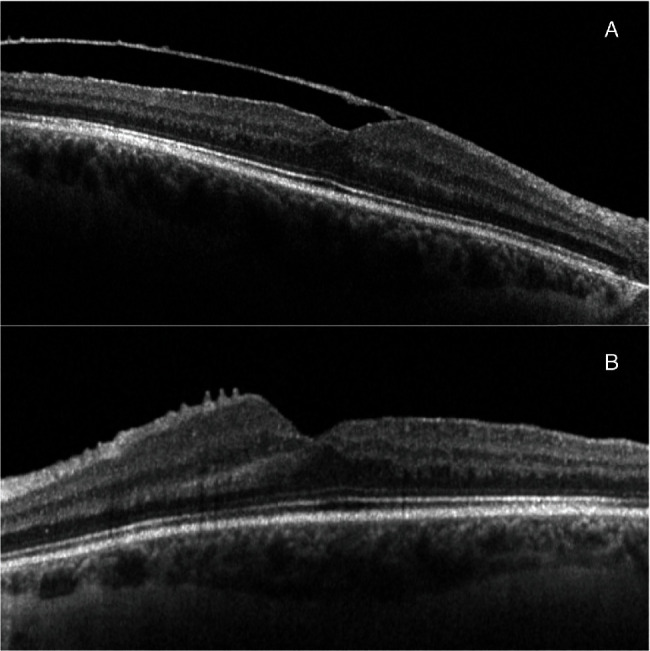
Optical coherence tomography one year after the surgery. Note: epiretinal membrane (ERM) formation with no foveal contour disturbance in the right eye (A) and irregularities of the inner retina in the left eye (B); outer retinal layers were preserved in both eyes.

The patient underwent a 25-gauge vitrectomy in both eyes. The surgery core and peripheral vitrectomy were done, followed by posterior vitreous detachment induction. After the removal of organized vitreous hemorrhage, the posterior pole was visualized, and golden crescent-shaped deposits consisting of small crystals were observed under the ILM: in the right eye anterior to the inferior temporal vascular arcade with the formation of fine ILM striae posterior to the inferior temporal vascular arcade and no foveal involvement (Figure 1(A)) and in the left eye posterior to the inferior temporal vascular arcade with no ILM striae formation(Figure 1(B)). Due to macular involvement in the left eye, an ILM peeling was performed (staining with ILM-blue dye for 60 seconds, DORC, Dutch Ophthalmic Research Center, International B.V., Zuidland, Netherlands) with sediment removal (Figures 2(A)–2(D)). At the end of the surgery, both eyes underwent 360-degree prophylactic peripheral endolaser and were left with balanced salt solution endotamponade. Ten days following the surgery, visual acuity improved to 6/20 on the right and 6/8.5 on the left. On the examination one year after the surgery, visual acuity in the right eye was 6/8.5 and 6/6 in the left eye, and optical coherence tomography showed epiretinal membrane (ERM) formation with no foveal contour disturbance in the right eye (Figure 3(A)) and irregularities of the inner retina with no clear ERM formation in the left eye (Figure 3(B)). The outer retinal layers were preserved in both eyes, but there was a bilateral relative thinning of the retinal layers in the temporal-to-foveal area, probably caused by compression from the weight of the sub-ILM hemorrhage.

## 3. Discussion and Conclusions

Common causes of sub-ILM hemorrhage include Valsalva retinopathy, ocular trauma, rupture of a macroaneurysm, and blood dyscrasia [3]. Terson syndrome is another etiology, believed to result from elevated retinal venous pressure and rupture of retinal capillaries leading to sub-ILM hemorrhages [3, 4]. The management options of sub-ILM hemorrhage can vary depending on multiple parameters, such as the general condition of the patient, visual acuity, bilaterality, location, severity of the hemorrhage, and time since its onset, associated ocular pathology [3, 5]. Treatment options could include observation, YAG laser hyaloidotomy, or pars plana vitrectomy with ILM peeling and aspiration of the underlying hemorrhage [5, 6]. In most cases of TS, spontaneous recovery is expected, and therefore, conservative treatment is usually opted for [1]. However, in cases with nonclearing vitreous hemorrhage, vitrectomy should be considered to recover vision and prevent epiretinal membrane formation [1]. The timing of the intervention can vary depending on the patient's general status, as well as the period between the accident and establishment of the diagnosis.

Synchysis scintillans or “cholesterolosis bulbi” is a very rare degenerative condition, associated with the accumulation of multiple golden-brown crystals in eyes with chronic retinal detachment, vitreous hemorrhage, or uveitis [7]. The time required for these crystals' formation remains unknown as is their exact etiology. According to the literature, the findings are usually concentrated in the vitreous cavity with or without the involvement of the anterior chamber and can be detected in patients from months to years following the relevant incident [7]. In this article, we report the novel finding of bilateral crystalline deposits accumulated under the internal limiting membrane in a patient with TS and long-standing intraocular hemorrhage, resembling the appearance of synchysis scintillans. Apparently, after the accident, our patient had a vitreous and concurrent subinternal limiting membrane hemorrhage. Findings supporting the hypothesis that the formation of crystalline deposits in our patient was secondary to sub-ILM hemorrhage is the presence of a “ring” sign usually formed in the cases with detached ILM secondary to massive hemorrhage [8]. Considering that his first ophthalmological examination occurred seven months after the accident, we believe that the subinternal limiting membrane hemorrhage underwent absorption and partial degeneration with the formation of a cholesterol-rich small, yellow deposits, as observed during the surgery. Interestingly, the findings were bilateral and crescent-shaped, with macular involvement in one eye but without in the other. The crescent-like appearance of the sediment may be explained by the anatomy and tension of the ILM as well as changes in composition caused by the long-term accumulation of the sub-ILM hemorrhage. Chemical composition analysis was not performed for these deposits, but characteristic reflectivity, color, and history of hemorrhage led us to formulate the “cholesterolosis” mechanism. While lipid- and protein-rich hard exudates, commonly seen in conditions such as diabetic retinopathy, hypertensive retinopathy, and Coats' disease, may bear a distant resemblance to the sediment described in this case, given our patient's history and ocular findings, as well as the sub-ILM location of the deposits, the most likely etiology appears to be Terson syndrome with an associated long-standing sub-ILM hemorrhage.

The timing of surgical intervention in cases of sub-ILM hemorrhage in patients with Terson syndrome is debatable [6]; our case shows that in instances where sub-ILM hemorrhage clearance is delayed, the formation of deposits can be observed, increasing the risk of secondary ERM formation and thinning of the retinal layers. Treatment options for sub-ILM deposits can vary, depending on the location of the sediment. If there is a macular involvement, we propose peeling of the internal limiting membrane and removal of the deposits, whereas in cases where the crystals are concentrated outside of the macula, follow-up can be considered.

## Data Availability

All data associated with this study are presented in the paper.

## Abbreviations

TS: Terson syndrome
sub-ILM: Subinternal limiting membrane.
